# 

**Published:** 1907-04

**Authors:** 


					﻿---------—--------------------------------------
A T~'| A X |T A	SUBSCRIPTION $1.00.
y I I y \|	1 I /\	MONTHLY.
>.Ve-r C'N Gt N-xA;*$ ,v i O.1
«” •«'»'•’ (DF MEDICINE
• Successor to Atlanta Medical and Surgical Journal, Established 1855.
~ anS Southern "Medical Record, Established 1870.
Vol. IX.	Atlanta, Ga., April, 1907.	No. 1.
				

